# Bisphenol-A and Female Infertility: A Possible Role of Gene-Environment Interactions

**DOI:** 10.3390/ijerph120911101

**Published:** 2015-09-07

**Authors:** Xiaona Huo, Dan Chen, Yonghua He, Wenting Zhu, Wei Zhou, Jun Zhang

**Affiliations:** 1 MOE-Shanghai Key Laboratory of Children’s Environmental Health, Xinhua Hospital, School of Medicine, Shanghai Jiao Tong University, 1665 Kong Jiang Road, Shanghai 200092, China; E-Mails: 15216652683@163.com (X.H.); simpledandan1981@163.com (D.C.); windy5490@163.com (W.Z.); zzsmile12@163.com (W.Z.); 2 School of Public Health, Guilin Medical University, Guilin 541004, China; E-Mail: hyhup3@hotmail.com; 3 School of Public Health, Shanghai Jiao Tong University, Shanghai 200025, China

**Keywords:** bisphenol-A, gene, epigenetics, female, infertility

## Abstract

*Background*: Bisphenol-A (BPA) is widely used and ubiquitous in the environment. Animal studies indicate that BPA affects reproduction, however, the gene-environment interaction mechanism(s) involved in this association remains unclear. We performed a literature review to summarize the evidence on this topic. *Methods*: A comprehensive search was conducted in PubMed using as keywords BPA, gene, infertility and female reproduction. Full-text articles in both human and animals published in English prior to December 2014 were selected. *Results*: Evidence shows that BPA can interfere with endocrine function of hypothalamic-pituitary axis, such as by changing gonadotropin-releasing hormones (GnRH) secretion in hypothalamus and promoting pituitary proliferation. Such actions affect puberty, ovulation and may even result in infertility. Ovary, uterus and other reproductive organs are also targets of BPA. BPA exposure impairs the structure and functions of female reproductive system in different times of life cycle and may contribute to infertility. Both epidemiological and experimental evidences demonstrate that BPA affects reproduction-related gene expression and epigenetic modification that are closely associated with infertility. The detrimental effects on reproduction may be lifelong and transgenerational. *Conclusions*: Evidence on gene-environment interactions, especially from human studies, is still limited. Further research on this topic is warranted.

## 1. Introduction

Female infertility is a complex disorder and can be caused by a number of factors including genetic, environmental and behavioral [1,2,3]. Since genomics at the population level are relatively stable over a short time span, environmental and lifestyle-related factors may play a more important role in the increase of infertility.

Endocrine disrupting chemicals (EDCs) are a class of chemicals, both natural and synthetic, that exist in the environment. They can interfere with the physiological function of the endocrine system [4] and adversely affect hormone balance by disrupting the secretion or regulation of hormones [5]. Therefore, EDCs have attracted more and more attention.

Bisphenol A (BPA), an EDC widely used as an industrial chemical, has estrogenic activity and was found in 95% of 394 adults urine samples from a reference population in the United States [6]. The European and US Food and Drug Administrations concluded that the current BPA levels may have no risk to the general population [7]. However, basic scientists contested that the entire population may suffer adverse health effects from current BPA levels [8,9,10]. Our review summarizes evidence on the association between BPA environmental exposure and female infertility, and focuses on the role of gene-environment interaction in this association.

## 2. Methods

A comprehensive search in PubMed was performed to identify relevant literature. The keywords included bisphenol-A, gene, epigenetics, female infertility, reproduction, and reproduction-associated keywords. All full-text articles and abstracts published in English prior to December 2014 were eligible for our review.

## 3. BPA and Its Exposure

BPA was first synthesized in 1891 [11]. The formula is (CH_3_)_2_C(C_6_H_4_OH)_2_ or C_15_H_16_O_2_ and the chemical name is 4,4’-dihydroxy-2,2-diphenyl propane [7]. It has been widely used in the manufacture of resins such as polycarbonate and epoxy. These chemicals are mainly used for the interior lacquer coating of food and beverage cans, thermal receipt paper, some dental sealants and fillings, detergents, soaps, lotions, shampoos, conditioners, and nail polishes [12,13].Over 3.5 million tons of BPA are produced a year worldwide and more than 100 tons are released into the atmosphere [14].

BPA is ubiquitous in the environment. Ingestion, inhalation and dermal absorption are the main routes of daily exposure [15]. The acceptable dosage of BPA, representing the safe exposure level in human, is ≤50 ug/kg/day [16]. In the general population, BPA has been detected in various tissues, including adult sera (0.2–20 ng/mL), placental tissues (11.2 ng/g), human breast milk (0.28–0.97 ng/mL), human colostrums (1–7 ng/mL), urine (1.12 ng/mL in women) [17], umbilical cord blood [18], saliva [19], follicular fluid (~1–2 ng/mL ), and amniotic fluid [20]. In adults BPA is mainly metabolized by the hepatic glucuronidation pathway. The biologic half-life of BPA is approximately 6 h, with almost complete excretion via urine in 24 h [21]. However, despite the rapid metabolism, BPA can accumulate in tissues for an extremely long time and experience a conjugation-deconjugation cycling. Thus, the excretion of partial BPA is delayed [22].

BPA can accumulate in reproductive organs [17] and act as an endocrine disruptor owing to its structural similarity to estrogen. It is a mixed agonist-antagonist to affect estrogens and other steroid hormones [23] such as antagonizing the hippocampal synaptogenesis induced by estrogens [24]. BPA could exert its impact at a very low dose. For example, BPA had estrogenic effects at 2 μg/kg [25] and may be more estrogenic *in vivo* [26]. Nevertheless, the estrogenic potency of BPA is 1000–100,000-fold less than that of estradiol [27].

Woodruff *et al.* [28] showed that the sensitive windows of human exposure to BPA include progestation (before, during and shortly after the formation of fertilized eggs), pregnancy, infancy, childhood and puberty. Fetuses are more vulnerable to the adverse effects of BPA due to an immature drug-metabolizing system [3].

## 4. BPA and Female Infertility

Infertility is generally defined as failure to achieve a successful pregnancy after 12 months or more of appropriate, timed unprotected intercourse [29]. It affects 10%–15% of couples [30]. In general, approximately 35% of infertility is due to female factors alone [31]. BPA can widely impact the female fertility through multiple pathways. More recent evidence demonstrated that BPA not only plays as “weak” estrogen, but also has many other potential biological activities [16].

### 4.1. BPA and Hypothalamus

BPA may cause developmental and/or functional deffects of the hypothalamic system, which could result in inability to achieve the reproductive capacity at puberty and maintain it during adulthood [32,33]. It was confirmed that irreversible alteration in hypothalamic-pituitary-gonadal axis caused by exposure to 500 μg/kg/day BPA in rats can lead to anovulation and infertility [34]. The underlying mechanism may invole the production of kisspeptin, a driving signal of gonadotropin release hormone (GnRH) secretion [35,36]. For example, newborn rats treated with BPA at the doses of 100 and 500 μg/day had significantly diminished levels of prepubertal*Kiss1* mRNA expression at the hypothalamus [37]. Paradoxically, Wang X *et al.* found that adult female mice treated with BPA (20 μg/kg) at proestrus could increase the *Kiss1* mRNA level. Such different regulatory actions of hypothalamic *Kiss1* system may be attributed to the sensitivity of the various components to the timing of treatment.

In addition, GnRH neurons themselves may be the target of EDC at the sensitive window (reviewed by Diamanti-Kandarakis *et al.*) [38]. For instance, prenatal BPA treated sheep (BPA 5 mg/kg/day in cotton seed oil from day 30 to 90 of gestation) showed a significant decrease in GnRH mRNA expression prior to the expected onset of preovulatory LH surge [39].

### 4.2. BPA and Pituitary

BPA could directly affect the gonadotropin synthesis. Brannick *et al.* [40] found that mice administered with 50 ug/kg/day of BPA had decreased levels of gonadotropin mRNA, Gnrhr and Nr5a1, key components of gonadotropin synthesis. Gnrhr is essential for signal transduction between the pituitary and the hypothalamus [41]. On the other hand, BPA also cause pituitary proliferation and an increase in gonadotroph number [40].

BPA may also disrupt *kiss1* system at the pituitary and affect gonadotropin release [35], suggested by findings that kisspeptin and GPR54 were co-expressed in rat gonadotrophs. Adult gonadectomized female rats supplemented with oestradiol showed an increase in Kiss1 mRNA levels but a decrease in GPR54 mRNA expression [42].

### 4.3. BPA and Ovary

BPA has various effects on the ovary. For instance, BPA exposure is associated with follicle loss [43]. It caused lower antral follicle counts [43], decreased oocyte survival [44], and even significant loss of primordial follicles by reducing ovarian follicular reserves in F3 generation females [45].

Polycystic ovary syndrome (PCOS) is the most common endocrinopathy of women of reproductive age. Its cardinal features are hyperandrogenism, insulin resistance and chronic anovulation [46]. The distinctive clinical features are hirsutism, menstrual disfunction and infertility [47]. Its etiology is not yet clear. Both human [48,49] and animal studies [34] have suggested a possible role of BPA in PCOS aetiopathogenesis. Recently, it was shown that women with PCOS had a significantly higher BPA level than women without PCOS [48,49]. Neonatal rats exposed to 500 μg/day BPA developed PCOS-like syndrome in adulthood [34].

BPA has also been shown to increase testosterone (T) concentration by stimulating ovaries to produce T [50] and inhibiting T hydroxylase activity [51]. Furthermore, it acts as a potent sex hormone-binding globulin binder, displaces androgens [51] and, therefore, increases serum free androgen index [49]. On the other hand, elevated androgen concentrations may down-regulate the UDP-glucuronosyltransferase activity and transcripts [52,53], resulting in a decrease in detoxification and clearance of BPA [34,54].

Additionally, BPA-exposure is linked to insulin resistance indices [49]. The impact of BPA on the development of insulin resistance was corroborated by animal data in that a single low dose of BPA (10 μg/kg) disturbed insulin action and glucose metabolism in adult mice [55].

### 4.4. BPA and Meiosis

Evidence shows that several key stages of oocyte development, including the onset of meiosis in the fetal ovary (prophase 1), perinatal follicle development, and adulthood resumption of meiosis are vulnerable to BPA [27]. In the fetal period, female mice exposed to low dose BPA (400 ng/day) had adverse effects on oogenesis by disrupting meiosis, resulting in synaptic defects and an increased rate of recombination. The perturbations caused chromosomally abnormal eggs and embryos when these fetuses reached adulthood [27]. Similar defects were observed in adult female rhesus macaques in that intrauterine exposure to BPA disrupted the meiotic prophase events by increasing the levels of recombination and centromeric associations between nonhomologous chromosomes [56]. An *in vitro* study of human fetal oocytes demonstrated that BPA was related to the disruption of meiotic maturation, spindle organization and chromosome alignment, and an increase in the rates of oocyte degeneration [57].

*In vivo* animal studies further demonstrated disturbances of BPA during the final stages of meiosis in adulthood. A study treated the juvenile females mice (20- to 22-day-old) with 20, 40, or 100 ng/g body weight/day BPA for 6–8 days and found a dose-related increase in the meiotic abnormalities, congressional failure and aneuploidy [58]. Pacchierotti *et al.* conducted acute (0.2 and 20 mg/kg for 1 day), sub-chronic (0.04 mg/kg for 7days) and chronic studies (0.5 mg/L in drinking water for 7 weeks) respectively. Only the chronic treatment group showed a significant increase in prematurely segregated chromatids. Eichenlaub-Ritter *et al.* repeated the subchronic exposure but failed to show a significant result. However, they observed an association between BPA and perturbed spindle morphology and lower rates of MII oocytes [59]. In the same vein, their *in vitro* study found that only high concentrations of BPA (10 ug/mL) induced congressional failure and meiotic arrest [59].

Similarly, human oocytes discarded by patients undergoing IVF/ICSI cycles were cultured *in vitro* with BPA (20, 200 ng/mL or 20 ug/mL). Oocytes were less likely to progress to MII and more likely to degenerate. Bipolar spindles and aligned chromosomes were significantly decreased among MII oocytes [57]. The BPA interference was dose-dependent. These findings may partly explain the decrease in fertility reported in the last decades. However, it is worth noting that the concentrations of BPA (20, 200 ng/mL or 20 ug/mL) in this study were much higher than the baseline BPA level (1–2 ng/mL) in human follicular fluid [20]. Further studies are needed to confirm whether an environmental relevant dose in accordance with the human natural exposure may cause adverse effects.

### 4.5. BPA and Oviduct

The main effect of BPA on the oviduct is progressive proliferative lesions (PPL). Newbold *et al.* treated neonatal mice with BPA (10, 100 or 1000 μg/kg/day) and found that all groups had PPL in the oviduct [60]. In another study, gestational mice were exposed to BPA at 0.1, 1, 10, 100, or 1000 μg/kg/day. PPL was observed in all groups [16].

### 4.6. BPA and Uterus

Animal studies have demonstrated that prenatal exposure of mice to BPA could elicit atypical hyperplasia and stromal polyps of the uterus [16] and endometriosis—like phenotype [61]. However, human epidemiological studies did not confirm these associations [62,63,64].

Additionally, experimental studies suggested that BPA exposure could impair the uterine receptivity [65,66,67], which is important for successful embryo implantation. A study showed that exposure of pregnant mice to BPA (0, 0.025, 0.5, 10, 40, and 100 mg/kg/day) during gestation days 0.5 to 3.5 resulted in defective uterine receptivity in the 100 mg/kg/day BPA-treated group [67]. Female mice exposure to BPA (6.75 and 10.125 mg/animal) on days 1–4 of gestation altered uterine morphology [68] and significantly reduced the number of implantation sites [69]. However, a very recent human epidemiological study did not support these results. Minguez-Alarcon *et al.* found no association between urinary BPA concentrations and *in vitro* fertilization (IVF) outcomes, including endometrial wall thickness, embryo quality, fertilization rates and implantation [70]. The discrepancy between animal and human findings may be attributed to BPA dose, exposure time and method between laboratory studies and epidemiological studies.

## 5. BPA-Gene Interaction

In the post genome era, gene-environment interactions have become a hot research topic [71]. Exposure to environmental factors, mainly nutritional, chemical and physical factors, potentially alters gene expression and changes the epigenome that can modify adult disease susceptibility and lead to disease phenotype [72,73].

While most previous animal and human studies examined the direct effect of BPA exposure on adverse pregnancy outcomes and infertility, little research has explored the role of BPA-gene interactions. Even the limited studies mostly focused on male rather than female infertility. Available evidence suggests that gene-environment interaction may be one of the major contributors to female infertility, and have lifelong [74] and transgenerational impact [75]. However, the mechanisms of gene-environment interactions are not yet fully clarified [72].

BPA was originally thought to work via binding to estrogen receptors (ERs) and triggering agonistic effects by mimicking hormonal action [76]. For instance, BPA selectively binds with classic ERs (ESR1 and ESR2) and acts as an ER modulator [77,78]. ESR1 and ESR2 play a significant role in the events of steroidogenesis, the growth of follicle, ovulation, and endometrial cycle [79]. In an *in vivo* mice study, BPA up-regulated mRNA expression of *ESR1* gene by 2.85-fold while the expression of *ESR2* gene showed no significant difference [80]. However, another *in vitro* study that exposed human fetal oocytes to BPA showed that the expression of *ESR2* gene was as up-regulated as *ESR1* and *ERRΓ* [81].

However, evidence from other systems now reveals that BPA can also affect gene expression directly and/or to impact epigenetic modification of fertility-related genes [82,83,84]. For example, in adult men, the gene expression of *ESR2* and *ESR1* increased in men with higher urinary BPA concentrations [85]. Caserta *et al.* [79] investigated 111 women aged 18–40 years, who were affected by primary infertility. The gene expression of nuclear receptors (*ESR1* and *ESR2*), androgen receptor (*AR*), pregnane X receptor (*PXR*), aryl hydrocarbon receptor (*AhR*), and peroxisome proliferator-activated receptor gamma (*PPARγ*) was analyzed as biomarkers in peripheral blood mononuclear cell. A positive correlation was found between BPA levels and *ESR1, ESR2, AR, AhR*, and *PXR* expression, while *PPARγ* expression did not show any meaningful difference (Table 1). These findings were confirmed in another small study [86] and supported the hypothesis that BPA acts on nuclear receptor (NR) through disturbing hormone response pathways and/or steroidogenesis [79] and, therefore, affects female infertility.

**Table 1:** BPA exposure and fertility related gene expression and genetic modification.

Author and Year	Species	Treatment Period	Dose	Tested Tissue	Change of Gene Expression
Caserta, *et al.* 2013 [79]	Infertile women	-	-	peripheral blood mononuclear cell	ESR1, ESR2,AhR, PXR up-regulation; AhR, PPARγ no difference
Chao, *et al.* 2012 [80]	Mice	Postnatal day 7–14 or 5–20	20–40ug/kg per day or per 5 days	Ovarian	ESR1 up-regulated; ESR2 no difference
				Oocytes	IGF 2Γ, PEG3 methylated sites decreased; H19 no difference
Brieno-Enriquez, *et al.* 2012 [81]	Human (fetuses)	Cultured 7–21 days	30umol/L	Cultured oocytes	Up-regulation: H2ax, Rpa, Spo11, ESR1, ESR2, ERRΓ, Blm at 14 day;No difference: Stra8, Nalp5, Smc1B, Sycp1
				Cultured fibroblasts	Up-regulation: H2ax, Rpa, Blm, ESR1, ESR2, ERRΓ, MIh1 at 21 day
Li, *et al.* 2014 [87]	Wistar rats	Postnatal day 28–35 day	10 or 40 mg/kg/day	Ovarian	FIGLA, H1FOO and AMH no difference
			160 mg/kg/day		FIGLA, H1FOO down-regulation; AMH up-regulation
Calhoun, *et al.* 2014 [88]	Rhesus Macaque	Gestational day 100–165	400 ug/kg/day deuterated BPA	Fetal uteri	Up-regulated: PDE11A, HOXC9, IGHMBP2, CSTL1, HOXC10, IL26, KLK3, ALX3, DOK6, ABHD1, HOXC6, HOXC8, HOXC9, HOXC10, HOXD1, HXOD3, HOXD9, WNT2, WNT4, WNT5A
					Down-regulated: CDH4, GDE1, GJB3, TFAP2C, RNF186, HOXA13, FGF10, CLIC6, CXCL14, SST, HOXA13
Susiarjo, *et al.* 2013 [89]	Mice	2 weeks prior to mating and embryonic (E) day 0–9.5	10 ug or 10 mg /kg/day	Placenta	LOI: Snrpn, Kcnq1ot1 Average total RNA (placentas with or without LOI) expression up-regulated: Snrpn, Kcnq1ot1; Placentas with LOI had higher RNA expression at lower dose: Snrpn; Average total RNA expression down-regulated: Cdkn1c (upper dose and lower dose), Ube3a; Methylation levels reduced: Snrpn, Kcnq1ot1
				Embryo	LOI: Igf2 Average total RNA expression up-regulated: Igf2; Methylation levels increased: Igf2 DMR1; Methylation levels reduced: H19/Igf2 ICR

Brieno-Enriquezet *et al.* [81] found that the expression of genes involved in double-strand break generation (*Spo11*), signaling (*H2ax*) and repair (*Rpa, Blm*) increased significantly in cultured fetal oocytes treated with BPA during meiotic prophase (Table 1). This possible molecular mechanism may explain the effects of BPA on female germ cells [81]. An *in vivo* animal study [90] focusing on the effect of BPA on the expression of meiosis-related genes treated pregnant mice with BPA (20 ng/g/day) beginning at 11 days postcoitus (dpc). Fetal ovaries were collected at 12-, 12.5-, 13.5-, and 14.5-day in pregnancy. Sixteen meiosis-specific genes were selected: meiotic entry gene *Stra8*; *Spo11*, *Sycp1*, *Sycp2*, *Sycp3*, *Syce1*, *Syce2* and *Tex12* (associated with the formation of synaptonemal complex); *Rec8*, *Stag3*, *Smc1b* (associated with sister chromatid cohesion), *Dmc1*, *Mei1*, *Msh4*, *and Msh5*, *Prdm9* (meiotic recombination pathway genes). All of these genes were up-regulated after BPA treatment for 3.5 days, although only *Msh4*, *Dmc1*, and *Sycp2* reached statistical significance. A dramatic increase in expression of all of the above genes was observed from 12 to 14.5 dpc. It raises the possibility that fetal BPA exposure may limit expansion of the primordial germ cell population.

Another *in vivo* rat study [87] showed a decrease in follicle number and an increase in constituent ratio of atretic follicles in relation to BPA levels. The alteration may be caused by the changed expression of follicle development-related genes such as *FIGLA, H1FOO* and *AMH* (Table 1) with increasing BPA doses. For example, compared with the 0 (control) group, the expression of *FIGLA* gene mRNA was significantly reduced in the 160mg/kg/day BPA group while the expression of *AMH* gene mRNA was significantly increased. However, *H1FOO* gene mRNA expression levels showed a significant decrease in all BPA groups. Whether these adverse effects and the potential mechanism are consistent with those in human needs further confirmation [87] as the dose used in this study was much higher than that of human exposure.

The expression levels of key development-related and functional genes of the uterus also have attracted great attention. When pregnant rhesus macaques were exposed to BPA at gestation day (GD) 100–165, significant differences in genes expression in fetal uteri were observed between BPA-exposed and placebo groups at GD165 (Table 1). It showed that BPA exposure in the third trimester could alter transcriptional signals and may, in turn, influence adult uterine function of the offspring. The article detailed the role of *HOXA13, WNT4* and *WNT5A*, which are critical for development and function of human reproductive organ [88]. The dose used in this study was 400 μg/kg/day, which resulted in unconjugated biologically active BPA of 0.3–0.5 ng/mL [56], which is similar to serum levels in human adults and fetuses [14].

Epigenetics may be another important mechanism of BPA-gene interaction in female infertility. The effects of epigenetic modifications generally include DNA methylation, histone modification (acetylation, methylation, phosphrylation, ubiquitination, sumoylation and ADP ribosylation), and expression of non-coding RNAs (including microRNA) [91]. Environmental toxicants may alter epigenome rather than DNA sequence [92]. Furthermore, the modification of the epigenome in the germ line might be transmitted to the progeny, therefore, promoting a transgenerational phenotype [92]. Chao *et al.* [80] evaluated the effects of BPA on the reprogramming of imprinted genes and found that the increased concentration of BPA remarkably decreased the methylation pattern of maternal imprinted genes (Table 1). Meanwhile, the expressions of four types of DNA methyltransferases (*Dnmt1*, *Dnmt3a*, *Dnmt3b and Dnmt3L*) were all suppressed with increasing BPA treatment concentrations. Another *in vivo* study [89] found maternal BPA exposure reduced the imprinted genes (*Snrpn*, *Ube3a*, *Kcnq1ot1*, *Cdkn1c*, *and Ascl2*) methylation levels in mice placentas with the dose of dietary BPA at 10 mg/kg/day. The RNA expression of these genes increased. While methylation levels of *Igf2* in embryos were up-regulated, the RNA expression was down-regulated at the dose of 10mg/kg/day BPA. Loss of imprinting (LOI) occurred at *Snrpn*, *Kcnq1ot1* and *Igf2* (Table 1). It was hypothesized that DNA methylation may regulate the imprinting, but perfect correlation was not found in this study [89]. They suggested that abnormal development of the placenta and embryo disturbed fetal and postnatal health. Thus, the epigenome is vulnerable to environmental perturbations, particularly during embryogenesis, neonatal development, and adolescence via epigenetic mechanisms.

## 6. Conclusions

To sum up, animal studies have demonstrated that BPA can affect female fertility by directly altering reproduction-related gene expression as well as impacting epigenetic modification. Critical and sensitive windows of susceptibility include progestation, pregnancy, infancy, childhood and puberty [28]. However, it should be pointed out that different species, or different development periods of the same species, different doses of BPA exposure, and/or different exposure modes and times, can all widely influence study results in animals. There are also significant variations between and within human populations. Literature on the association between BPA and female infertility and its potential biological mechanisms is still limited. Evidence on gene-environment interaction is even more scarce. More research on this topic is warranted.
